# Influenza infection as a trigger for systemic lupus erythematosus flares resulting in hospitalization

**DOI:** 10.1038/s41598-021-84153-5

**Published:** 2021-02-25

**Authors:** Young Bin Joo, Ki-Jo Kim, Kyung-Su Park, Yune-Jung Park

**Affiliations:** Division of Rheumatology, Department of Internal Medicine, College of Medicine, St. Vincent’s Hospital, The Catholic University of Korea, Seoul, Republic of Korea

**Keywords:** Rheumatology, Risk factors

## Abstract

In patients with systemic lupus erythematosus (SLE), there are concerns that infections may increase the risk of flares. We evaluated the association between influenza infection and SLE flares resulting in hospitalization. SLE flares resulting in hospitalization and influenza cases were ascertained from the Korean national healthcare insurance database (2014–2018). We used a self-controlled case series design. We defined the risk interval as the first 7 days after the influenza index date and the control interval was defined as all other times during the observation period of each year. We estimated the incidence rates of SLE flares resulting in hospitalization during the risk interval and control interval and compared them using a Poisson regression model. We identified 1624 influenza infections among the 1455 patients with SLE. Among those, there were 98 flares in 79 patients with SLE. The incidence ratio (IR) for flares during the risk interval as compared with the control interval was 25.75 (95% confidence interval 17.63–37.59). This significantly increased the IRs for flares during the risk interval in both women (IR 27.65) and men (IR 15.30), all age groups (IR 17.00–37.84), with and without immunosuppressive agent (IR 24.29 and 28.45, respectively), and with and without prior respiratory diseases (IR 21.86 and 26.82, respectively). We found significant association between influenza infection and SLE flares resulting in hospitalization. Influenza infection has to be considered as a risk factor for flares in all SLE patients regardless of age, sex, medications, and comorbidities.

## Introduction

Systemic lupus erythematosus (SLE) is an autoimmune disease wherein patients often experience repeated disease flares during the disease course. These flares are related to poor outcomes; it has been reported that severe flares^1,2^ and the number of flares^3^ are associated with accrual of irreversible organ damage, which links to mortality^4–6^. Thus, prevention of flares is an important target in the management of SLE.

There are several known risk factors for disease flares such as ultraviolet^7–9^, infections^7,10–12^, and hormones^7,13,14^. Among the risk factors for disease flares, some environmental factors such as ultraviolet and infections could be modified with effort by the patient. For example, patients with SLE are recommended to use sunblock to avoid ultraviolet exposure. In addition, the risk of infection such as influenza or pneumococcus can be reduced through vaccination and personal hygiene management.

Influenza is a frequently occurring infection; however, unlike for other viruses such as herpes and cytomegalovirus, influenza vaccination has been developed and are recommended annually in people with high risk. Exposure to influenza could easily occur in patients with SLE and may increase the risk of SLE flares^15^. The concept that the immune response to viral infection is different in patients with SLE than in healthy individuals supports the possible triggering effect of the influenza virus on SLE flares. For example, the type I interferon (IFN) system is activated to defend against viral infection in healthy individuals and is normally terminated after eradicating the pathogen^16^. However, a dysregulated type I IFN system in SLE stimulates continuous production of IFN through the presence of endogenous nucleic acids, which could increase disease activity in SLE^16^.

Infections have been associated with a higher risk of SLE flares, but the impact of influenza infection on SLE flares has not been evaluated. Therefore, we conducted an assessment in a lupus cohort.

## Results

### Baseline characteristics of the patients in the study

**Figure 1:**
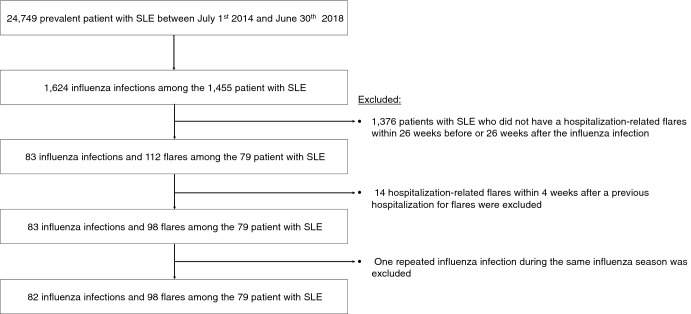
Patient selection flow chart. *SLE* systemic lupus erythematosus.

There was a total of 24,749 prevalent patients with SLE in the database between July 2014 and June 2018. Of them, 1455 patients with SLE experienced 1624 influenza infections. After the exclusion criteria were applied, 79 patients with SLE who had 83 influenza infections and 98 SLE flares resulting in hospitalization during observational period were included in the analysis according to the inclusion and exclusion criteria. The flow chart showing patient selection according to the exclusion criteria is provided in Fig. 1.

The mean age of patients with SLE with flares resulting in hospitalization was 35.7 (SD 14.9) years and 68 (86.1%) were women (Table 1). Information regarding medications, comorbidities, previous SLE flares, and previous respiratory disease of the patients is presented in Table 1.

**Table 1:** Baseline characteristics of 79 patients with SLE.

Characteristic	Value
Number of patients	79
**Age, year**	
Mean	35.7 ± 14.9
**Age group**	
< 20	12 (15.2)
20–29	18 (22.8)
30–39	18 (22.8)
40–49	18 (22.8)
≥ 50	13 (16.5)
**Sex**	
Women	68 (86.1)
Men	11 (13.9)
**Immunosuppressive agents**^**a**^	
At least one immunosuppressive agent listed below	68 (86.1)
Intravenous cyclophosphamide	5 (6.3)
Mycophenolate mofetil	22 (27.9)
Methotrexate	3 (3.8)
Azathioprine	6 (7.6)
Cyclosporine	5 (6.3)
Tacrolimus	11 (13.9)
Hydroxychloroquine	42 (53.2)
Glucocorticoid	67 (84.8)
Dose, mg/day	
≤ 5	31 (46.3)
> 5, ≤ 10	13 (19.4)
> 10, ≤ 20	17 (25.4)
> 20	7 (10.5)
**Comorbidities**	
CCI index, mean value	1.96 ± 1.60
CCI index 0	14 (17.7)
1–2	39 (49.4)
≥ 3	26 (32.9)
**Previous respiratory diseases during the 6 months prior to hospitalization**^**b**^	
Yes	17 (21.5)
No	62 (78.5)

Values are presented as N (%) or mean ± standard deviation.

Table shows the information at the time of the first flares during study period among the 79 patients with SLE who have experienced a total of 98 SLE flares.

*SLE* systemic lupus erythematosus, *CCI* Charlson comorbidity index.

^a^Immunosuppressive agents which have been prescribed during 6 months before hospitalization.

^b^Respiratory disease codes were extracted from International Classification of Disease (ICD)-10 codes (I27.8, I27.9, J40.x-J47.x, J60.x-J67.x, J68.4, J70.1, J70.3) for Charlson comorbidity index analysis.

#### Incidence ratios for SLE flares resulting in hospitalization after influenza infection

The estimated incidence ratio (IR) for SLE flares resulting in hospitalization during the risk interval as compared with the control interval was 25.75 (95% confidence interval 17.63–37.59) (Table 2). As we divided the risk interval into days 1–3 and days 4–7, the IR for SLE flares resulting in hospitalization was 21.81 (14.71–32.35) and 7.56 (3.69–15.47), respectively. That is, the effect of influenza infection on SLE flares resulting in hospitalization is stronger in the early period (days 1–3) after the influenza infection, but a similar positive association was seen in the later period (days 4–7) as well, although its effect is smaller than that in the early period.

**Table 2:** Incidence ratios for SLE flares resulting in hospitalization after influenza infection.

Risk interval	Incidence ratio	95% CI
During risk interval for 7 days/control interval	25.75	17.63–37.59
Days 1–3/control interval	21.81	14.71–32.35
Days 4–7/control interval	7.56	3.69–15.47

*SLE* systemic lupus erythematosus, *CI* confidence interval.

### Sensitivity analysis

As wash-out period prior to SLE flares resulting in hospitalization was extended to 8 weeks or 12 weeks, the estimated IR for SLE flares resulting in hospitalization was 30.08 (19.98–45.29) and 28.76–18.79 44.01), which was similar to those using 4 weeks wash-out period (Table 3). Sensitivity analyses according to different control interval (preexposure or postexposure 25 weeks) and different definitions of SLE flares and influenza infection were conducted. The estimated IR for SLE flares resulting in hospitalization during the risk interval as compared with the postexposure control interval was 26.02 (22.07–30.69) and that compared with the preexposure control interval was 24.81 (20.45–30.10). As the SLE flare was limited to the use of glucocorticoid ≥ 30 mg or ≥ 50 mg, the estimated IRs for SLE flares resulting in hospitalization were not different (IR 23.54 vs. 24.95, ≥ 30 mg vs. ≥ 50 mg, respectively). In the case that influenza infection was defined with diagnosis code or medication code, the result was not different to the case wherein the influenza infection was defined with only the medication code (IR 22.73).

**Table 3:** Sensitivity analysis of incidence ratios for SLE flares resulting in hospitalization after influenza infection.

Variable	Incidence ratio (95% CI)
**Wash out period prior to hospitalization**	
8 weeks	30.08 (19.98–45.29)
12 weeks	28.76 (18.79–44.01)
**Control interval**	
Preexposure 26 weeks	24.81 (20.45–30.10)
Postexposure 25 weeks	26.02 (22.07–30.69)
**SLE flares**	
Use of glucocorticoid ≥ 30 mg/day	23.54 (9.43–58.77)
Use of glucocorticoid ≥ 50 mg/day	24.95 (13.67–45.55)
**Influenza infection defined with**	
Diagnosis code or medication code	22.73 (16.37–31.57)

*SLE* systemic lupus erythematosus, *CI* confidence interval.

### Subgroup analysis

In both women and men, the IR for SLE flares resulting in hospitalization was higher in the risk interval than in the control interval (IR 27.65 vs. 15.30, women vs men, respectively) (Table 4). The effects of influenza infection on SLE flares resulting in hospitalization were not different according to age group and the use of immunosuppressive agent. Presence or absence of previous respiratory disease during the 6 months prior to hospitalization was not associated with an increased or decreased risk for SLE flares resulting in hospitalization after influenza infection.

**Table 4:** Subgroup analysis of incidence ratios for SLE flares resulting in hospitalization after influenza infection.

Subgroup	Incidence ratio (95% CI)
**Sex**	
Women	27.65 (18.38–41.62)
Men	15.30 (5.23–44.76)
**Age group**	
< 20	25.50 (11.10–58.50)
20–29	27.20 (11.92–62.07)
30–39	23.54 (9.43–58.77)
40–49	17.00 (5.98–48.35)
≥ 50	37.84 (19.52–73.36)
**Use of immunosuppressive agent**^**a**^	
Yes	24.29 (14.87–39.67)
No	28.45 (15.76–51.33)
**Use of corticosteroid**^**a**^	
Yes	22.30 (14.77–33.67)
No	59.50 (21.82–162.23)
**Comorbidities**	
CCI index 0	35.06 (18.41–66.76)
≥ 1	20.04 (12.95–31.02)
**Previous respiratory diseases during the 6 months prior to hospitalization**^**b**^	
Yes	21.86 (9.59–49.84)
No	26.82 (17.50–41.11)

*SLE* systemic lupus erythematosus, *CCI* Charlson comorbidity index.

^a^Immunosuppressive agents or steroids which have been prescribed during 6 months before hospitalization.

^b^Respiratory disease codes were extracted from International Classification of Disease (ICD)-10 codes (I27.8, I27.9, J40.x-J47.x, J60.x-J67.x, J68.4, J70.1, J70.3) for Charlson comorbidity index analysis.

## Discussion

Using the longitudinal data from the population-based cohort, we examined the impact of the influenza infection on SLE flares resulting in hospitalization. We found a significant impact with influenza infection on increased SLE flares resulting in hospitalization within seven days after influenza infection.

The self-controlled case series design is proposed to investigate the associations between acute outcomes and transient exposures involving only cases who have experienced the outcome of interest^17^. One of the main advantages of the case series method is that it controls the confounders that do not vary with time over the observational periods, such as genetics, socio-economic status, and severity of underlying disease^17^. Thus, the self-controlled case series design, which is adjusted for factors mentioned above, is useful to identify the association between SLE flares (acute outcome) and influenza infection (transient exposure) using claims data rather than nested case–control design.

SLE flares as an outcome of interest in the study was focused in the case with hospitalization for SLE activity. The SELENA SLEDAI^18^ shows the items that indicate severe flare. Among them, an increase in the prednisone dose to > 0.5 mg/kg/day or hospitalization indicated severe flare. Considering the factors suggested in the SELENA SLEDAI, only the cases who have SLE code as a main diagnostic code on admission as well as who have been treated with moderate to high doses of steroids were selected in the analysis. Regarding steroid dosage, we defined a steroid dose ≥ 20 mg/day as a component of flares considering moderate to severe flares. Instead, we conducted sensitivity analysis using different steroid doses such as ≥ 30 mg/day or ≥ 50 mg/day. All the results according to different dosage of steroid in the sensitivity analysis showed similar results as those of the steroid dose ≥ 20 mg/day.

This study has some limitations. First, the influenza infection was not confirmed by a laboratory test but by the prescription code for oseltamivir phosphate. Therefore, in the design of our study, it is very important to ensure that patients prescribed oseltamivir phosphate are real patients with influenza infection. If patients were prescribed oseltamivir phosphate for flu-like symptoms without an influenza laboratory test, but the flu-like symptoms were found to be symptoms in the early stage of lupus flare, incidence rate of “flare triggered by flu” may have been miscalculated. In South Korea, however, it is common practice in the medical institutions to perform an influenza diagnostic test prior to oseltamivir phosphate prescription. South Korea has a different medical system than other countries, and people with influenza infection can easily access physicians and be prescribed oseltamivir phosphate. Rapid result report of influenza diagnostic test (10 min to 2 h) and reasonable cost of influenza diagnostic test let both patients and doctors conduct the influenza diagnostic test prior to oseltamivir phosphate prescription. In addition, Korean insurance guidelines are presented to prescribe oseltamivir in influenza confirmed patients or strongly suspected patients who have certainly respiratory symptoms except fever. Fever is a common symptom but respiratory symptoms are not common in early stage of lupus flare. The possibility that patients prescribed oseltamivir phosphate do not have real influenza infection is very low in Korean medical circumstance.

Second, we did not consider intravenous peramivir as a medication code for influenza. Peramivir is not covered by insurance and is therefore not identified in claims data in South Korea. However, peramivir is usually prescribed as a second line medication or is limited to patients who find it difficult to take medicine. Nevertheless, it is possible that patients with influenza treated with peramivir were missing.

Considering these two limitations, we further conducted sensitivity analysis with influenza infection defined using a diagnosis code or medication code, which showed a significant association between influenza infection and SLE flares resulting in hospitalization as the main result in the study.

Lastly, adherence to medication, which is one of the possible risk factors for flares, was not considered due to limitation of claims data. Compared to the case–control study design, however, this effect is thought to be small as this study was conducted in only cases.

In conclusion, we found a significant association between influenza infection and SLE flares resulting in hospitalization. Possibility of increased risk for SLE flares resulting in hospitalization within seven days after influenza infection has to be considered when treating and educating patients with SLE.

## Methods

### Ethics statement

This study protocol was approved by the Institutional Ethics Review Board (IRB) of St. Vincent’s Hospital, Catholic University of Korea (VC17ZESI0103). All methods were performed in accordance with the relevant guidelines and regulations. The need for written informed consent was waived by the IRB of St. Vincent’s Hospital, Catholic University of Korea because data were de-identified and collected retrospectively.

### Data source

A population-based SLE prevalent cohort from July 2014 to June 2018 was constructed using the Korean national healthcare insurance (KNHI) database. All people in South Korea are eligible for coverage under the KNHI Program. This database contains individual information about healthcare service usage (diagnostic code, length of stay, treatment costs, services received) and prescription records (drug code, days prescribed, daily dosage).

#### Definitions of SLE, SLE flares resulting in hospitalization, and influenza infection

SLE, SLE flares, and influenza infection were identified using the diagnostic code or/and prescription code or/and medical care utilization code in the KNHI database.

The diagnosis of SLE was made based on the diagnostic code for both SLE International Classification of Disease (ICD)-10 code M32 and rare intractable disease (RID) code V136. The RID is a registration system operated by the KNHI. Only SLE patients who fulfil the 1997 American College of Rheumatology (ACR)^19^ or the 2012 Systemic Lupus International Collaborating Clinics (SLICC)^20^ classification criteria are registered in the RID system by physicians, mainly rheumatologists.

As the outcome, SLE flares resulting in hospitalization was defined based on two components. That is, among the SLE patients with both ICD-10 code M32 and RID code V136, SLE flares resulting in hospitalization was defined in the cases that were both hospitalized for more than two days and prescribed corticosteroids ≥ 20 mg/dl for more than one day.

Influenza infection was defined in the cases that have prescription code for oseltamivir phosphate, which is a medication for influenza treatment (358901ACH, 358902ACH, 358903ACH, 358907ACH, 358907ASS, 358908ASS, 358909ASS, 358910ASS). The index date is the first day to be prescribed oseltamivir phosphate in the influenza season. As influenza tends to occur during autumn to spring and to peak in winter, each period of influenza season in the study was defined from July in the year to June in the next year (e.g. Jul 2014 to Jun 2015 or Jul 2016 to Jun 2017).

### Inclusion and exclusion criteria

We included the SLE patients who fulfilled the SLE operational definition and have experienced influenza infection after being diagnosed with SLE. Among the patients with SLE treated from July 1st 2014 to June 30th 2018, patients with SLE who did not have any SLE flares resulting in hospitalization within 26 weeks before or 26 after influenza infection were excluded. Moreover, SLE flares resulting in hospitalization within 4 weeks after a previous hospitalization for flares were excluded as the repeated flares within a short period could be an incomplete cure of the first flare. Lastly, only one patient who had repeated influenza infection during the same influenza season was excluded (the gap between first and second influenza index date was 38 days).

### Study design

**Figure 2:**
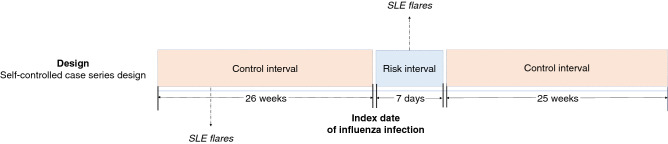
Study design. This study was conducted using the self-controlled case series design. The index date of influenza infection is the first day to be prescribed oseltamivir phosphate. The number of SLE flares resulting in hospitalization are counted on a yearly (52 weeks) basis. Risk interval was defined as the first 7 days from the index date of influenza infection. The control interval was defined as all other times during the observation period of each influenza season. *SLE* systemic lupus erythematosus.

This is a historic cohort study and was conducted to investigate the association between influenza infection (a transient exposure) and SLE flares (an acute outcome). To exclude the effects of possible confounding factors on the outcome of interest, self-controlled case series design was used in this study (Fig. 2). The risk interval was defined as the first 7 days from the index date of influenza infection. The control interval was defined as all other times during the observation period. In the case that the index date of influenza infection is January 1st 2016, for example, the risk interval is from January 1st to January 7th and the control interval is from July 1st 2015 to June 30th, except for the 7 days of risk interval. The control interval was divided into the preexposure and postexposure control interval. Preexposure control interval means for the 26 weeks before the influenza index date and postexposure means for the 25 weeks after the last day of risk interval.

### Statistical analysis

Based on a self-controlled case series analysis, which could investigate a transient exposure and an acute outcome within only “case” individuals who have experienced the outcome of interest with fixed effects covariates^17^, we estimated the incidence rates of SLE flares resulting in hospitalization during risk interval and control interval and compared them using a fixed-effects conditional Poisson regression model. The model allows auto-correlation and can be used in the data having multiple exposure and outcome occurrences^21,22^.

A sensitivity analysis further conducted to confirm the robustness of our results. First, wash-out period prior to SLE flares resulting in hospitalization was extended to 8 weeks or 12 weeks to ensure that each hospitalization is a new episode of flare, not a partially treated prior episode. Second, control interval was limited to preexposure 26 weeks or postexposure 25 weeks to identify that previous influenza infection have remote effect on lupus flares. Third, SLE flares resulting in hospitalization were limited to the use of corticosteroid ≥ 30 mg or ≥ 50 mg. Lastly, influenza infection was defined as cases with either the influenza diagnosis code or oseltamivir phosphate medication code.

We performed subgroup analysis according to the age group (< 16 years, 16–49 years, and ≥ 50 years), sex, use of immunosuppressive agents or glucocorticoids, dose of glucocorticoid, and comorbidities based on the Charlson comorbidity index. Immunosuppressive agents included cyclophosphamide, mycophenolate mofetil, methotrexate, azathioprine, cyclosporine, tacrolimus, and hydroxychloroquine.

All statistical analyses were performed using SAS software (version 9.4; SAS Institute Inc., Cary, NC, USA). A p value < 0.05 was considered statistically significant.
